# *Chrysanthemum boreale* Makino Inhibits Oxidative Stress-Induced Neuronal Damage in Human Neuroblastoma SH-SY5Y Cells by Suppressing MAPK-Regulated Apoptosis

**DOI:** 10.3390/molecules27175498

**Published:** 2022-08-26

**Authors:** Parkyong Song, Seo Young Choi, Ji Sun Hwang, Hyeon Cheal Park, Keun Ki Kim, Hong-Joo Son, Chang-Oh Hong, Yu-Jin Kim, Wanil Kim, Kwang Min Lee

**Affiliations:** 1 Department of Convergence Medicine, Pusan National University School of Medicine, Yangsan 50612, Korea; 2 Department of Life Science and Environmental Biochemistry, Life and Industry Convergence Research Institute, Pusan National University, Miryang 50463, Korea; 3 New Drug Development Center, Daegu–Gyeongbuk Medical Innovation Foundation, K-MEDI Hub, Daegu 41061, Korea; 4 Department of Biochemistry, Department of Convergence Medical Science, Institute of Health Sciences, Gyeongsang National University College of Medicine, Jinju 52727, Korea

**Keywords:** *Chrysanthemum boreale* Makino, oxidative stress, hydrogen peroxide, cell death, cell survival, neurodegenerative diseases

## Abstract

Oxidative stress has been demonstrated to play a pivotal role in the pathological processes of many neurodegenerative diseases. In the present study, we demonstrated that *Chrysanthemum boreale* Makino extract (CBME) suppresses oxidative stress-induced neurotoxicity in human neuroblastoma SH-SY5Y cells and elucidated the underlying molecular mechanism. Our observations revealed that CBME effectively protected neuronal cells against H_2_O_2_-induced cell death by preventing caspase-3 activation, Bax upregulation, Bcl-2 downregulation, activation of three mitogen-activated protein kinases (MAPKs), cAMP response element-binding protein (CREB) and NF-κB phosphorylation, and iNOS induction. These results provide evidence that CBME has remarkable neuroprotective properties in SH-SY5Y cells against oxidative damage, suggesting that the complementary or even alternative role of CBME in preventing and treating neurodegenerative diseases is worth further studies.

## 1. Introduction

Reactive oxygen species (ROS) are chemically reactive molecules normally produced in living organisms as a result of normal cellular metabolism [1]. ROS plays important roles in mediating cellular activities, such as immune responses, inflammation, cell survival, and stress responses. However, owing to their high reactivity, excess production of ROS can lead to harmful responses, including cell death or oxidative stress, which results from an imbalance in pro-oxidant/antioxidant homeostasis [2]. Among various ROS, H_2_O_2_ is one of the major agents produced by oxidative stress and acts as a precursor of ROS. H_2_O_2_ induces apoptosis in various cells, and neural cells exposed to H_2_O_2_ undergo necrosis and apoptosis [3,4]. Cumulative oxidative stress may induce cellular damage and mitochondrial dysfunction, which are major factors in aging and neurodegenerative diseases, such as Alzheimer’s disease and Parkinson’s disease. Because of the high oxygen demand of the brain, it is susceptible to the effects of high levels of ROS, and protection from oxidative stress is crucial for the prevention and treatment of neurodegenerative disorders. Several recent studies have reported that inhibiting ROS generation and increasing cellular antioxidants may be beneficial for protecting against neurodegenerative diseases caused by oxidative stress [5]. Therefore, antioxidants are thought to be substances that may protect against brain disorders. Natural products such as polyphenols, flavonoids, carbohydrates, and their derivatives contain a large group of antioxidant compounds with reducing and radical scavenging properties [6].

*Chrysanthemum boreale* Makino, belonging to the Asteraceae family, is a small, yellow-flowered wild species widely distributed in East Asian countries including mainland China, South Korea, and Japan [7,8,9,10,11]. It is an important medicinal plant that contains various pharmacologically active components including polyacetylenes, essential oils, and flavonoids [12,13,14,15]. *C. boreale* has been reported to possess potential medicinal properties against bacterial action, inflammation, angiogenesis, and hypertension [16,17,18,19,20,21,22]. However, there is no scientific evidence of the neuroprotective properties of *C. boreale*. Therefore, in this study, we investigated the effect of *C. boreale* on oxidative stress-induced cell damage in human neuroblastoma SH-SY5Y cells. 

## 2. Results

### 2.1. Chrysanthemum boreale Makino Extract Exhibited No Obvious Cytotoxic Effects in SH-SY5Y Cells

**Figure 1:**
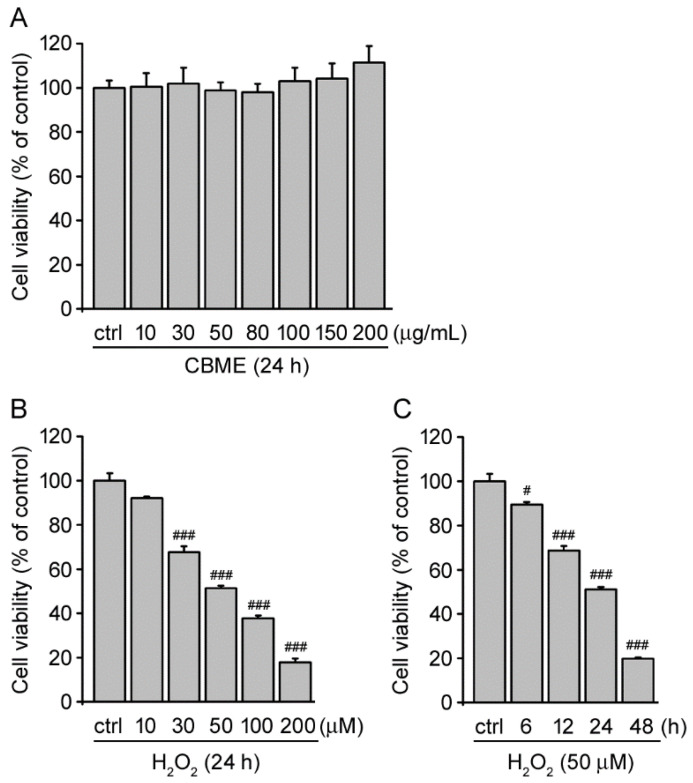
Effect of *C. boreale* Makino extract (CBME) and hydrogen peroxide (H_2_O_2_) on cell viability in SH-SY5Y cells. (**A**) The cells were exposed to the indicated doses of CBME for 24 h. Cell viability was evaluated using a WST-1 assay. (**B**) The cells were exposed to the indicated concentrations of H_2_O_2_ for 24 h. Cell viability was evaluated using the WST-1 assay. (**C**) The cells were incubated with 50 µM H_2_O_2_ for the indicated durations. Cell viability was evaluated using the WST-1 assay. The results are representative of four independent experiments. All data are presented as the mean ± SEM. # *p* < 0.05, ### *p* < 0.005, significantly different from the control group.

Initially, we examined the toxicity of *C. boreale* Makino extract (CBME) on SH-SY5Y human neuroblastoma cells. The cell viability assay showed that CBME did not exert any apparent cytotoxic and proliferative effects at concentrations of up to 200 µg/mL in SH-SY5Y cells under normal conditions (Figure 1A). Therefore, we used CBME concentrations of 50, 100, and 150 µg/mL to evaluate the effect of CBME against oxidative stress in a dose-dependent manner.

We treated SH-SY5Y cells with different concentrations of hydrogen peroxide (H_2_O_2_) for 24 h to determine the suitable concentration at which its effects on cytotoxic potency can be observed. The viability of SH-SY5Y cells decreased in a concentration-dependent manner. At a concentration of 50 µM, H_2_O_2_ resulted in ~50% cell mortality (Figure 1B). The cytotoxic potency of H_2_O_2_ at 50 µM concentration was confirmed at different incubation durations ranging from 6 to 48 h (Figure 1C). Thus, cells were treated with 50 µM H_2_O_2_ for 24 h for subsequent experiments.

### 2.2. Chrysanthemum boreale Makino Extract Inhibited Hydrogen Peroxide (H_2_O_2_)-Induced Neurotoxicity in SH-SY5Y Cells

**Figure 2:**
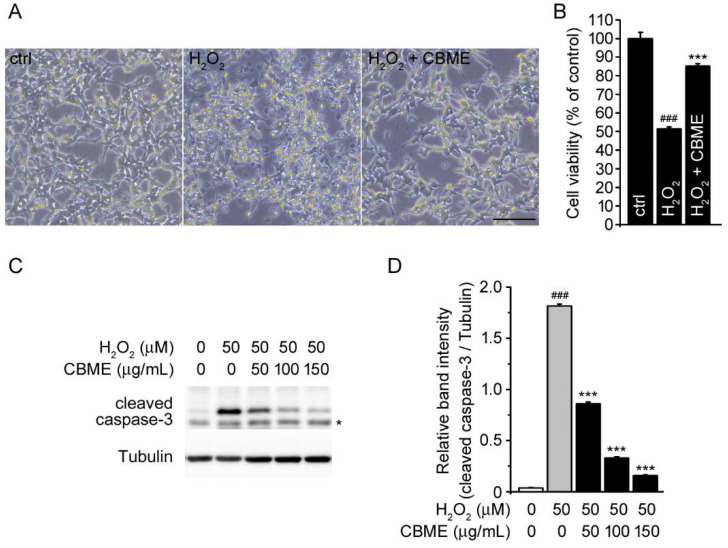
Effects of *Chrysanthemum boreale* Makino extract (CBME) on H_2_O_2_-induced neural damage in SH-SY5Y cells. (**A**) Microscopic image of SH-SY5Y cells with or without 3 h pretreatment with CBME (150 µg/mL) and subsequently exposed to 50 µM H_2_O_2_ for an additional 24 h. The representative image was obtained by phase-contrast microscopy. Scale bar = 100 µm. (**B**) Cell viability was evaluated using the WST-1 assay in the same conditions as (**A**). (**C**) Western blot bands corresponding to cleaved caspase-3 in SH-SY5Y cells which were pre-treated with the indicated concentrations of CBME for 3 h and then exposed to H_2_O_2_ for 24 h (* denotes non-specific band). Tubulin was used as the loading control. (**D**) Relative band intensity of cleaved caspase-3 was quantified using densitometric analysis and then normalized to that of tubulin. Values in the bar graphs are the mean ± SEM of at least three independent experiments. *** *p* < 0.005, significantly different from the H_2_O_2_ group. ### *p* < 0.005, significantly different from the control group.

To investigate the neuroprotective effects of CBME, we performed the following experiment using an oxidative stress model in which H_2_O_2_ was used to treat SH-SY5Y human neuroblastoma cells. As expected, we observed H_2_O_2_-induced alterations in cell morphology as reported previously (Figure 2A) [23,24]. The cells which were only treated with H_2_O_2_ underwent massive cell death, whereas the CBME pre-treated cells were resistant to cell death, which was confirmed both visually and by the WST-1 assay (Figure 2A,B).

H_2_O_2_, a well-known substance generated by oxidative stress, can evoke neuronal cell death by acting as a mediator of apoptosis [25,26,27,28,29]. Caspase-3, a member of the cysteine-aspartic acid protease (caspase) family, executes apoptosis and has been shown to be activated by H_2_O_2_ as a principal apoptosis-associated effector caspase [30,31,32,33]. Therefore, we evaluate caspase-3 activity by measuring the level of cleaved caspase-3 protein using Western blotting to confirm the protective effect of CBME on H_2_O_2_-induced oxidative stress. When the cells were treated with only H_2_O_2_, the level of cleaved caspase-3 was increased compared with that in vehicle-treated cells (Figure 2C, first and second lanes). However, the cells pre-treated with CBME showed a significant decrease in the level of cleaved caspase-3 in a CBME concentration-dependent manner against the H_2_O_2_-induced expression of cleaved caspase-3 (Figure 2C,D). These results showed that CBME protected SH-SY5Y cells against H_2_O_2_-induced cell damage.

### 2.3. Chrysanthemum boreale Makino Extract Inhibited H_2_O_2_-Stimulated Bax/Bcl-2 Pathway in SH-SY5Y Cells

**Figure 3:**
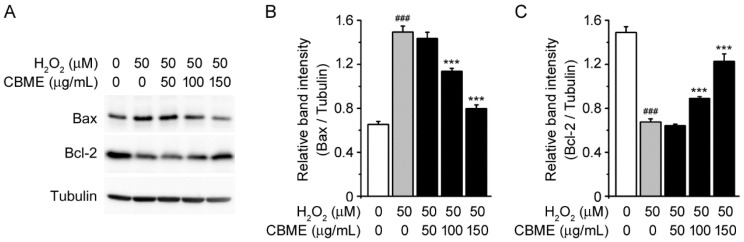
Effects of *Chrysanthemum boreale* Makino extract (CBME) on Bax and Bcl-2 expression in SH-SY5Y cells exposed to hydrogen peroxide. (**A**) Western blot bands corresponding to Bax and Bcl-2 in SH-SY5Y cells which were pre-treated with the indicated concentrations of CBME for 3 h and then exposed to H_2_O_2_ for 24 h. Tubulin was used as the loading control. (**B**) Relative band intensity of Bax were quantified using densitometric analysis and then normalized to tubulin. (**C**) Relative band intensity of Bcl-2 was quantified using densitometric analysis and then normalized to that of tubulin. Values in the bar graphs are the mean ± SEM of at least three independent experiments. *** *p* < 0.005, significantly different from the H_2_O_2_ group. ### *p* < 0.005, significantly different from the control group.

As pathological apoptosis is induced by various apoptosis-associated genes [34], we further investigated the neuroprotective effect of CBME against H_2_O_2_-induced cell death by measuring the expression levels of pro-apoptotic Bax and anti-apoptotic Bcl-2 proteins [35]. In cells treated with H_2_O_2_ alone, the level of pro-apoptotic Bax protein was increased, whereas the level of anti-apoptotic Bcl-2 protein was decreased in comparison with that in vehicle-treated cells (Figure 3B, first and second lanes). However, pretreatment with CBME decreased H_2_O_2_-stimulated Bax expression and increased H_2_O_2_-reduced Bcl-2 expression in a dose-dependent manner (Figure 3B,C). These data confirmed that CBME effectively protected neuronal cells against H_2_O_2_-induced cell death.

### 2.4. Chrysanthemum boreale Makino Extract Suppressed H_2_O_2_-Induced Mitogen-Activated Protein Kinase (MAPK) Pathway

Numerous studies have demonstrated that H_2_O_2_-induced oxidative stress may trigger apoptosis by activating the MAPK family [36,37,38]. Therefore, we examined the involvement of the MAPK pathway in the mechanism underlying this action to understand the molecular mechanism underlying the suppression of H_2_O_2_-induced cell damage by CBME. The activation of ERK 1/2, JNK 1/2, and p38, which are members of the MAPK kinase family, was tested by measuring the level of phosphorylated proteins.

**Figure 4:**
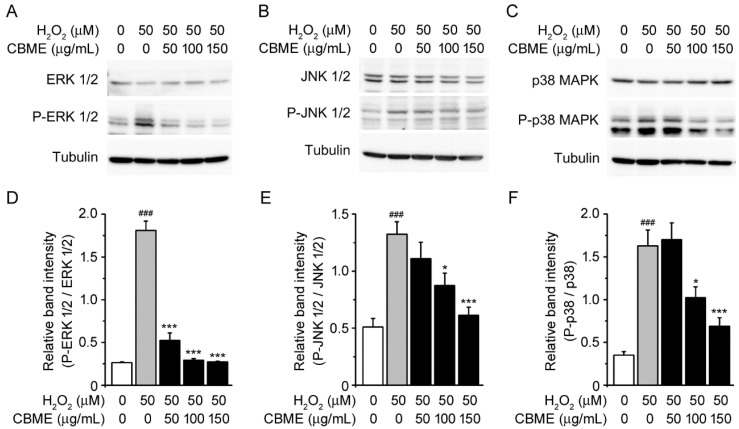
Effects of *Chrysanthemum boreale* Makino extract (CBME) on the MAPK pathway in SH-SY5Y cells exposed to H_2_O_2_. (**A**) Western blot bands corresponding to ERK 1/2 and P-ERK 1/2 in SH-SY5Y cells, which were pre-treated with the indicated concentrations of CBME for 3 h and then exposed to H_2_O_2_ for 24 h. Tubulin was used as the loading control. (**B**) Western blot bands corresponding to JNK 1/2 and P-JNK ½. (**C**) Western blot bands corresponding to p38 and P-p38. (**D**) Relative band intensity of P-ERK 1/2 was quantified using densitometric analysis and then normalized to that of ERK 1/2. (**E**) Relative band intensity of P-JNK 1/2 was quantified using densitometric analysis and then normalized to that of JNK 1/2. (**F**) Relative band intensity of P-p38 was quantified using densitometric analysis and then normalized to that of p38. Values in the bar graphs are the mean ± SEM of at least three independent experiments. *** *p* < 0.005, * *p* < 0.05, significantly different from the H_2_O_2_ group. ### *p* < 0.005, significantly different from the control group.

H_2_O_2_ treatment significantly increased the levels of phospho-ERK 1/2, phospho-JNK 1/2, and phospho-p38 MAPK proteins compared with vehicle (Figure 4A–C, first and second lanes). However, incubation with CBME decreased H_2_O_2_-induced phosphorylation of JNK 1/2, p38, and especially ERK 1/2 (Figure 4) in a dose-dependent manner. The total protein levels of the three MAPKs did not change in H_2_O_2_- and/or CBME-treated cells. These observations indicate that CBME protects neuronal cells against H_2_O_2_-induced oxidative stress by inhibiting the activation of MAPK signaling.

### 2.5. Chrysanthemum boreale Makino Extract Prevented H_2_O_2_-Induced Phosphorylation of CREB in SH-SY5Y Cells

**Figure 5:**
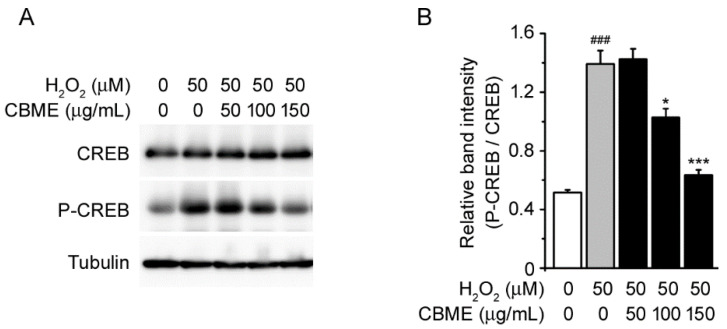
Effects of *Chrysanthemum boreale* Makino extract (CBME) on CREB phosphorylation in SH-SY5Y cells exposed to H_2_O_2_. (**A**) Western blot bands corresponding to CREB and P-CREB in SH-SY5Y cells, which were pre-treated with the indicated concentrations of CBME for 3 h and then exposed to H_2_O_2_ for 24 h. Tubulin was used as the loading control. (**B**) Relative band intensity of P-CREB was quantified using densitometric analysis and then normalized to that of CREB. Values in the bar graphs are the mean ± SEM of at least three independent experiments. *** *p* < 0.005, * *p* < 0.05, significantly different from the H_2_O_2_ group. ### *p* < 0.005, significantly different from the control group.

CREB, a cAMP response element-binding protein, is an important transcription factor with various functions [39,40] and is known to play an essential role in neuronal survival [41,42] and long-term memory formation [43,44,45]. CREB activation occurs via phosphorylation of serine 133 by a number of protein kinases, including MAPKs [46,47]. To further explore the neuroprotective mechanism of CBME in SH-SY5Y cells, we investigated the phosphorylation of CREB protein using Western blotting analysis. We observed that CREB phosphorylation was enhanced by H_2_O_2_ treatment (Figure 5A, first and second lanes). However, the enhanced phosphorylation of CREB was suppressed by incubation with CBME at concentrations of 100 and 150 µg/mL (Figure 5A,B). These results suggest that CBME has the potential to inhibit H_2_O_2_-induced cell death by suppressing MAPK-regulated apoptosis.

### 2.6. Chrysanthemum boreale Makino Extract Attenuated H_2_O_2_-Induced NF-kB Phosphorylation and iNOS Induction

**Figure 6:**
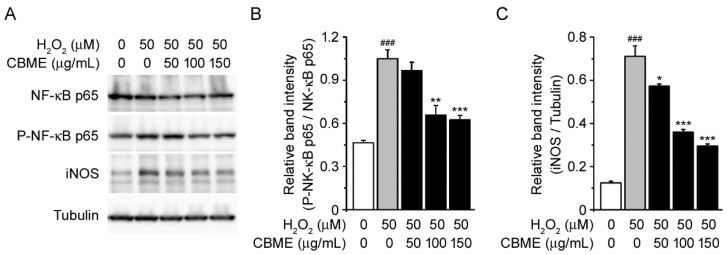
Effects of *Chrysanthemum boreale* Makino extract (CBME) on NF-κB phosphorylation and iNOS in SH-SY5Y cells exposed to H_2_O_2_. (**A**) Western blot bands corresponding to NF-κB, P- NF-κB, and iNOS in SH-SY5Y cells, which were pre-treated with the indicated concentrations of CBME for 3 h and then exposed to H_2_O_2_ for 24 h. Tubulin was used as the loading control. (**B**) Relative band intensity of P-NF-κB was quantified using densitometric analysis and then normalized to that of NF-κB. (**C**) Relative band intensity of iNOS was quantified using densitometric analysis and then normalized to that of tubulin. Values in the bar graphs are the mean ± SEM of at least three independent experiments. *** *p* < 0.005, ** *p* < 0.01, * *p* < 0.05, significantly different from the H_2_O_2_ group. ### *p* < 0.005, significantly different from the control group.

Next, we evaluated the protective molecular mechanisms of CBME by measuring inflammation-related protein expression. The NF-κB pathway is a pivotal pro-inflammatory mediator that plays a key role in oxidative stress [48,49,50]. Therefore, we examined the protein levels of NF-κB, phospho-NF-κB, and iNOS in H_2_O_2_-induced SH-SY5Y cells. After H_2_O_2_ exposure, the protein expression of phospho-NF-κB and iNOS was significantly upregulated compared with that in vehicle-treated cells (Figure 6A, first and second lanes). However, the increased phosphorylation of NF-κB was attenuated by incubation with CBME at concentrations of 100 and 150 µg/mL. Moreover, iNOS expression was suppressed by CBME in a dose-dependent manner (Figure 6A,B). The total protein levels of NF-κB did not change in H_2_O_2_-and/or CBME-treated cells. These results suggest that CBME protects SH-SY5Y cells against H_2_O_2_-induced oxidative stress by attenuating NF-κb-mediated inflammation.

## 3. Discussion

Oxidative stress has been demonstrated to play a pivotal role in the pathological processes of many neurodegenerative diseases including Alzheimer’s disease, Parkinson’s disease, and amyotrophic lateral sclerosis [51,52,53]. Natural products have less toxicity and fewer side effects as compared to chemical compounds. They can help in preventing and treating various neurodegenerative diseases. For this reason, numerous studies have attempted to investigate their neuroprotective properties which can be leveraged to inhibit neural damage caused by oxidative stress [54,55,56,57].

In the present study, we investigated the protective activity of CBME in human neuroblastoma SH-SY5Y cells from H_2_O_2_-induced apoptosis. Numerous studies have used SH-SY5Y cells as a cellular model for research on H_2_O_2_-induced neurotoxicity [58,59]. H_2_O_2_ is widely known as one of the major inducers of oxidative stress, resulting in neuronal cell death [60], and a link between neurodegenerative diseases and apoptosis and inflammation in the brain has also been reported [61,62]. Based on our results, we demonstrated that CBME pretreatment significantly attenuated neuronal cell injury caused by H_2_O_2_-induced oxidative stress (Figure 2A,B). Moreover, we confirmed the protective effects by measuring the level of cleaved caspase-3 protein (Figure 2C,D), a critical executor of apoptotic cell death that is activated by H_2_O_2_ [33,63].

The anti-apoptotic factor Bcl-2 and pro-apoptotic factor Bax are well characterized as being related to cell viability [64]. Moreover, H_2_O_2_-induced apoptosis has been shown to have a functional relationship with the anti- and pro-apoptotic regulatory factors Bcl-2 and Bax in neurons [65]. Overexpression of Bcl-2 heterodimerizes with Bax to repress apoptotic cell death. In contrast, Bax enhances apoptosis in response to a variety of death signals (such as, DNA damage, growth factor withdrawal, steroid hormones, or ligation of death receptors) [66,67]. Our data showed that CBME repressed Bax expression and enhanced Bcl-2 expression during H_2_O_2_-induced apoptosis (Figure 3). Thus, these results demonstrate the protective role of CBME in the regulation of Bcl-2- and Bax-dependent apoptotic pathways.

**Figure 7:**
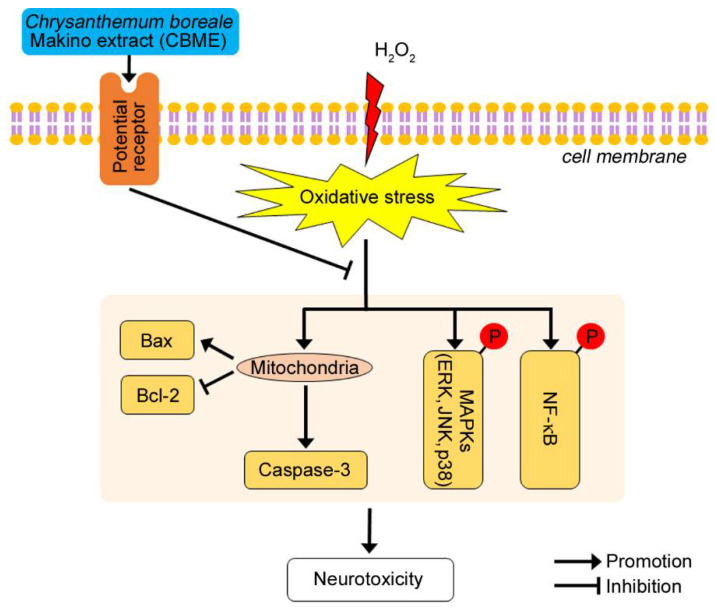
A schematic diagram illustrating the proposed protective mechanism of CBME against oxidative stress. CBME protects neuronal cells against H_2_O_2_-induced cell death by preventing caspase-3 activation, Bax upregulation, Bcl-2 downregulation, MAPK-regulated apoptotic cell loss, and NF-κB-mediated inflammation.

We also examined the possible association of the MAPK pathway to elucidate the molecular mechanisms underlying the suppression of H_2_O_2_-induced cell damage by CBME. We observed that the phosphorylation levels of JNK 1/2, p38, and ERK 1/2 were markedly increased in H_2_O_2_-treated cells as previously reported (Figure 4A–C, first and second lanes) [24,68,69,70,71]. Interestingly, CBME pretreatment significantly suppressed H_2_O_2_-induced phosphorylation of JNK 1/2, p38, and especially ERK 1/2 (Figure 4A,D). A feasible explanation is that CBME may be the most sensitive to trigger caspase-3 inactivation and ERK 1/2 inactivation, allowing a degree of neuroprotection against H_2_O_2_-induced cell damage. Reportedly, ERK phosphorylates CREB [72,73]. To further explore the neuroprotective mechanism of CBME in SH-SY5Y cells, we investigated the phosphorylation of CREB protein (Figure 5) because the phosphorylation of ERK was dramatically reduced by CBEM in the aforementioned results (Figure 4A,D). Moreover, we observed inflammation-related protein expression (Figure 6) because increased phosphorylation levels of JNK and p38 can further lead to inflammatory reactions [74,75,76,77]. Our results also showed that after H_2_O_2_ exposure, the phosphorylation of CREB and NF-κB, and iNOS expression was significantly elevated. Notably, these effects were reversed in a dose-dependent manner by CBME treatment. These results strongly support the hypothesis that CBME has the potential to suppress H_2_O_2_-induced cell death by attenuating MAPK-regulated apoptotic cell loss and NF-κB-mediated inflammation. The proposed neuroprotective mechanism of CBME is shown in Figure 7.

Taken together, we showed that CBME protected against neurotoxicity from H_2_O_2_-induced oxidative stress, and the protective mechanism was concomitant, at least partly, through the MAPK-mediated pathway, as well as caspase-3 inactivation. Our current findings provide the first evidence that CBME has remarkable neuroprotective properties in SH-SY5Y cells against oxidative damage and the various mechanisms involved. Although further studies are necessary to examine it’s in vivo functions and explain further details of the underlying mechanisms, this suggests that CBME may have a potential therapeutic effect on oxidative stress-related neurological disorders. In conclusion, the present study suggests that CBME should be studied further as a complementary or even an alternative role in preventing and treating neurodegenerative diseases.

## 4. Materials and Methods

### 4.1. Cell Culture

Human neuroblastoma SH-SY5Y cells were obtained from the Korean Cell Line Bank (Seoul, Korea) and cultured in Dulbecco’s modified Eagle’s medium (GIBCO, Waltham, MA, USA) supplemented with 10% heat-inactivated fetal bovine serum (Hyclone, Logan, UT, USA), penicillin (100 units/mL), and streptomycin sulfate (100 µg/mL). Cells were maintained at 37 °C in a humidified atmosphere containing 5% CO_2_. Cells were pretreated with various concentrations of CBME for 3 h and then washed twice with phosphate-buffered saline (PBS). Subsequently, these pre-treated cells were exposed to H_2_O_2_ (50 µM) for 24 h.

### 4.2. Preparation of Chrysanthemum boreale Makino Extract

The plant extract powder (code number: KPM032-046) used in this study was obtained from the Korea Plant Extract Bank at the Korea Research Institute of Bioscience and Biotechnology (Daejeon, Korea) [78]. Briefly, whole dried portions of *C. boreale* Makino were cut into small pieces and extracted with 99.9% methyl alcohol for 3 days. After extraction, the solutions were filtered, concentrated, and dried to obtain a powder. The extract powder was dissolved in dimethyl sulfoxide before use.

### 4.3. Cell Viability Assay

Cell viability after H_2_O_2_ or CBME treatment was measured using the water-soluble tetrazolium salt 1 (WST-1; EZ-CyTox, Dogen, Korea) assay. SH-SY5Y cells were seeded into 96-well plates at a density of 5 × 10^4^ cells/well, and incubated in Dulbecco’s modified Eagle’s medium for 24 h. The cells were treated with the indicated concentration of either H_2_O_2_ or CBME for the indicated duration, and then cell viability was measured using the WST-1 assay according to the manufacturer’s instructions. After the WST-1 solution was added to each well (10% of the total volume), and the cells were incubated for 1 h, the absorbance was measured using a microplate reader at 450 nm. The optical density of the control cells was considered 100% viability.

### 4.4. Immunoblot Analysis

Proteins were separated by sodium dodecyl sulfate-polyacrylamide gel electrophoresis and transferred onto polyvinylidene fluoride membranes. After blocking with 3% BSA in TBS-T (137 mM NaCl, 20 mM Tris-Cl, pH 7.6, 0.1% Tween 20), the membranes were incubated with various primary antibodies, including anti-cleaved caspase-3 (cell signaling 9664), anti-Bax (cell signaling 2772), anti-Bcl-2 (cell Signaling 15071), anti-p44/42 mitogen-activated protein kinase (MAPK; Erk1/2) (R&D systems MAB1576), anti-phospho-p44/42 MAPK (Erk1/2) (cell signaling 4377), anti-p38 MAPK (cell signaling 8690), anti-phospho-p38 MAPK (cell signaling 4511), anti-JNK (cell signaling 9252), anti-phospho-JNK (cell signaling 4668), anti-CREB (cell signaling 9197), anti-phospho-CREB (cell signaling 9198), anti-NF-κB (cell signaling 8242), anti-phospho-NFkB (cell signaling 3033), anti-iNOS (cell signaling 13120), and anti-tubulin (Sigma-Aldrich T6199). The blots were then incubated with a secondary antibody [anti-rabbit horseradish peroxidase-conjugate or anti-mouse horseradish peroxidase conjugate (Santa Cruz Biotechnology)], and the protein bands were visualized using an enhanced chemiluminescence detection system (Bio-Rad).

### 4.5. Statistical Analysis

Significant differences between groups were determined using two-tailed unpaired Student’s *t*-tests, and multiple comparisons were performed using one-way or two-way repeated-measures ANOVA with Tukey’s post-tests. The analysis was performed using Origin 8.0 (OriginLab Corporation, Northampton, MA, USA). Data are expressed as the mean ± standard error of the mean (SEM) of at least three independent experiments. *p* values < 0.05 were considered statistically significant and are indicated in the figure legends.

## Data Availability

Not applicable.
